# Hypertonic saline for traumatic brain injury: a systematic review and meta-analysis

**DOI:** 10.1186/s40001-022-00897-4

**Published:** 2022-11-20

**Authors:** Nafiseh Gharizadeh, Morteza Ghojazadeh, Amirreza Naseri, Sanam Dolati, Faezeh Tarighat, Hassan Soleimanpour

**Affiliations:** 1 Research Center for Evidence-Based Medicine, Iranian EBM Centre: A Joanna Briggs Institute (JBI) Center of Excellence, Tabriz University of Medical Sciences, Tabriz, Iran; 2 Student Research Committee, Tabriz University of Medical Sciences, Tabriz, Iran; 3 Physical Medicine and Rehabilitation Research Center, Aging Research Institute, Tabriz University of Medical Sciences, Tabriz, Iran; 4 Emergency and Trauma Care Research Center, Tabriz University of Medical Sciences, Golgasht Street, Tabriz, Iran

**Keywords:** Brain injuries, Traumatic, Hypertonic solutions, Saline solution, Hypertonic, Systematic review, Meta-analysis

## Abstract

**Background:**

Traumatic brain injury (TBI) causes mortality and long-term disability among young adults and imposes a notable cost on the healthcare system. In addition to the first physical hit, secondary injury, which is associated with increased intracranial pressure (ICP), is defined as biochemical, cellular, and physiological changes after the physical injury. Mannitol and Hypertonic saline (HTS) are the treatment bases for elevated ICP in TBI. This systematic review and meta-analysis evaluates the effectiveness of HTS in the management of patients with TBI.

**Methods:**

This study was conducted following the Joanna Briggs Institute (JBI) methods and PRISMA statement. A systematic search was performed through six databases in February 2022, to find studies that evaluated the effects of HTS, on increased ICP. Meta-analysis was performed using comprehensive meta-analysis (CMA).

**Results:**

Out of 1321 results, 8 studies were included in the systematic review, and 3 of them were included in the quantitative synthesis. The results of the meta-analysis reached a 35.9% (95% CI 15.0–56.9) reduction in ICP in TBI patients receiving HTS, with no significant risk of publication bias (*t*-value = 0.38, df = 2, *p*-value = 0.73). The most common source of bias in our included studies was the transparency of blinding methods for both patients and outcome assessors.

**Conclusion:**

HTS can significantly reduce the ICP, which may prevent secondary injury. Also, based on the available evidence, HTS has relatively similar efficacy to Mannitol, which is considered the gold standard therapy for TBI, in boosting patients' neurological condition and reducing mortality rates.

**Supplementary Information:**

The online version contains supplementary material available at 10.1186/s40001-022-00897-4.

## Introduction

Traumatic brain injury (TBI) is the most common cause of mortality and long-term disability among young adults [1]. It is estimated that about seventy million individuals suffer from TBI each year [2], which makes it a consequential public health concern worldwide and imposes a significant cost on the healthcare system [3]. Road traffic injuries, falls, and violence are among the most common cause of TBI [4].

The first physical hit is not the only injurious mechanism in TBI. Biochemical, cellular, and physiological changes after the physical injury, which is called secondary injury [5], are significantly associated with poor neurological outcomes and mortality in these patients [6]. Studies suggested that increased intracranial pressure (ICP), which is a common complication associated with TBI [7], is a factor associated with secondary injury in TBI patients [8].

Hyperosmolar therapy, such as Mannitol and Hypertonic saline (HTS), is one of the primary treatment bases for elevated ICP in TBI [9, 10]. Several insights have been gained about HTS and mannitol in TBI. HTS is considered routine care in TBI patients [11]. Several studies assessed the efficacy of hyperosmolar components in decreasing ICP and overall outcomes of patients with TBI, but there are still controversies in this regard [12, 13]. In a meta-analysis of randomized controlled trials (RCTs) in 2016, HTS was compared with any other solutions in severe TBI. This study found no significant difference between HTS and other solutions in lowering mortality or improving ICP [14]. Also, a recent Cochrane review in 2020, based on weak available evidence, found that HTS is no better than mannitol in TBI patients [15].

These mentioned reviews did not include recent publications. In addition, observational studies were not included in these studies. This systematic review and meta-analysis evaluates the efficacy of HTS in the management of elevated ICP secondary to TBI, as the primary outcome. The effects of HTS in lowering mortality rates and improving neurological outcomes are also investigated as secondary outcomes.

### Methods

This systematic review was completed following the methods reported in Joanna Briggs Institute (JBI) *Manual for Evidence Synthesis* [16] and Preferred Reporting Items for Systematic Reviews and Meta-Analyses (PRISMA) statement [17].

### Eligibility criteria

Studies, that assessed the effects of HTS in any concentration and dosage, on ICP in patients with TBI were included in this systematic review. Non-English papers, review articles, commentaries, letters, and these were not included.

### Search

A systematic search was conducted in Medline via PubMed, EMBASE, Scopus, ProQuest, Google Scholar, and Web of Science in February 2022 with no limitations. The details of search strategies are presented in Additional file 1.

### Study selection

The results of database searches were imported into EndNote × 9 software and after removing the duplicated results, two independent researchers (NG, SD) assessed the meeting eligibility criteria in two title/abstract and full-text stages. Disagreements in the study selection process were resolved through consultation or by referring to another author (HS), who is an expert in this topic.

### Data collection

Data extraction was conducted using an electronic table in Microsoft excel which included the following parameters: the name of the first author of the study, the publication year, study design, setting of the study, mean age of the participants, the male ratio, assessed interventions, mortality rate, neurological outcomes, and outcomes about ICP.

### Risk of bias assessment

The risk of bias in RCTs was assessed using the JBI checklist [18]. JBI critical appraisal tool for RCTs assesses the risk of bias regarding the randomization, allocation concealment, the similarity of the groups in the baseline, blinding of participants and researchers, identic received treatment (other than the intervention of interest), follow-up completion, analyzing the participants in the groups to which they were randomized, identic and reliable outcomes measurement, and statistical analysis. For cross-sectional studies [19], quasi-experimental studies [18], and case–control studies [19], the relevant checklists were utilized.

### Data synthesis

Meta-analysis was performed using the comprehensive meta-analysis (CMA) software [20] with mean and SD for changes in ICP (in percent) by HTS. A random effect model was utilized for the meta-analysis. 95% confidence intervals (CIs) and 0.05 level of significance for *p*-value were observed and the result was presented as the forest plot. Also, the publication bias was assessed using the Begg and Mazumdar's correlation test [21] and presented as the funnel plot.

## Results

### Study inclusion

**Fig. 1:**
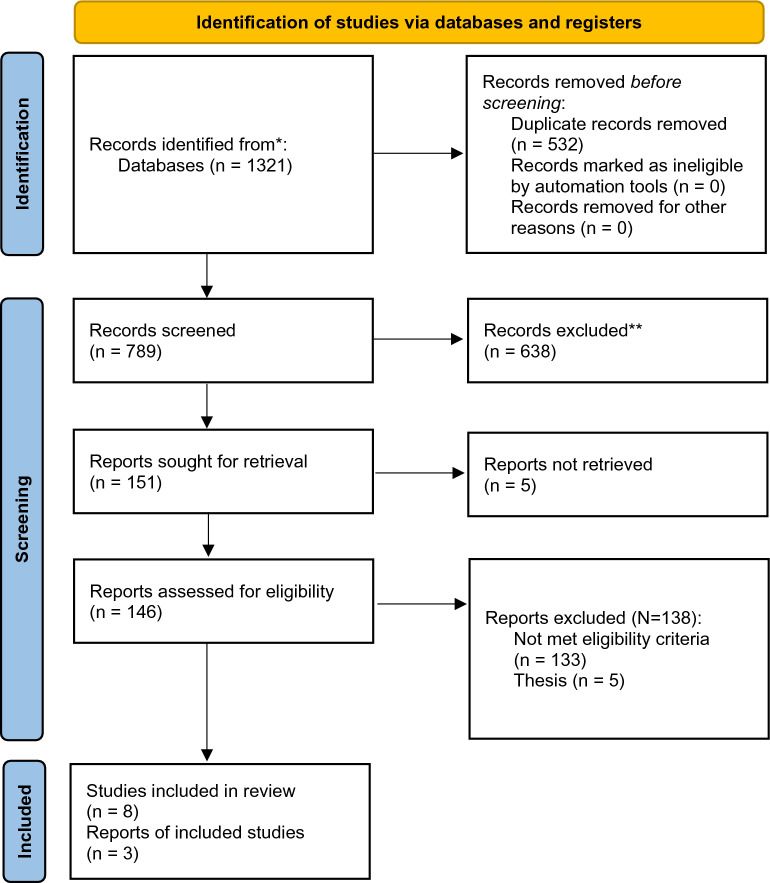
PRISMA flow diagram

The details of the selection process are presented in the PRISMA flow diagram (Fig. 1). In summary, out of 1321 results of databases searching, 8 studies were included in this systematic review [22–29] and 3 of them were included in the quantitative synthesis. Three of these studies were observational studies and the rest five studies had a clinical trial study design. The publication years were 1998 to 2018. The sample size in these studies was between 6 and 60 and the mean age of the participants varies between 30 and 55. 90-day neurologic status was reported in one study [25], Cottenceau reported a 6-month follow-up, and finally, and Jagannatha et al. only assessed this outcome over 6 days. Table 1 shows a summary of the characteristics and findings of the included studies.

**Table 1:** The characteristics and results of the included studies

Study	Design	Setting	Participants	Age	Male ratio	Intervention	Mortality	Neurologic outcome	ICP
Ware M. 2005	Retrospective study	San Francisco General Hospital, United States	13	42 ± 15	76.9%	22 treatments with 23.4% HTS and 19 treatments Mannitol	–	–	ICP peak: Mannitol: 38 mm Hg HTS: 36 mm Hg average reduction in ICP: Mannitol: 20 mm Hg HTS: 15 mm Hg
Francony G. 2008	RCT	Michallon’s Hospital, Grenoble, France	20 (Mannitol: 10 and HTS: 10)	Mannitol: 43 ± 11 HTS: 37 ± 16	Mannitol: 70% HTS: 90%	100 mL of 7.45% HSS and 231 mL of 20% mannitol	–	–	Mannitol: 45% reduction ICP HSS: 32% reduction ICP
Vialet R. 2003	RCT	University hospital trauma center, France	20 (Mannitol: 10 and HTS: 10)	Mannitol: 30.8 ± 19 HTS: 35.0 ± 18	Mannitol: 40% HTS: 50%	20% mannitol (1160 mOsm/kg/H2O) or HTS: (2400 mOsm /kg/H2O)	Mannitol: 50% HTS: 40%	Severe GOS: Mannitol: 50% HTS: 60%	Number of episodes per day ICP < 25 mm Hg: Mannitol: 13.3 ± 14.2 HTS: 6.8 ± 5.5 Total duration of episodes ICP < 25 mm Hg: 95 ± 92 Mannitol: HTS: 62 ± 81
Cheng F. 2018	Retrospective study	First People’s Hospital of Kunshan, China	60 (Mannitol: 30 and HTS: 30)	Mannitol: 41.53 ± 15.27 HTS: 42.27 ± 17.03	Mannitol: 83.3%HTS: 80%	3% HTS or 20% mannitol	2 HTS vs. 1 mannitol; P = 0.554	–	Mean daily ICP burden: Mannitol: 12.37 ± 2.95 HTS: 11.57 ± 3.65
Cottenceau V. 2011	RCT	two university hospitals from France and Israel	47 (Mannitol: 25 and HTS: 22)	Mannitol: 36.1 ± 16.8 HTS: 42.7 ± 19.9	–	7.5% saline or 20%manitol	No significant difference in Glasgow Outcome Scales	–	ICP after 30 min: Mannitol: 10.5 ± 6.8 HTS: 12.2 ± 6.1 ICP after 120 min: Mannitol: 13.6 ± 7.5 HTS: 13.9 ± 7.8
Jagannatha AT. 2017	RCT	United Kingdom	38 (Mannitol: 20 and HTS: 18)	Mannitol: 31 ± 13 HTS: 27 ± 8	Mannitol: 90% HTS: 88%	20% mannitol or 3% saline, in an equimolar dose	Favorable GOS score at 6 months: Mannitol: 0 HTS: 2	In-hospital mortality: Mannitol: 10 HTS: 3 6 months mortality: Mannitol: 10 HTS: 6	Fall in ICP (mmHg): Mannitol: 8.9 ± 8.4 HTS: 10.1 ± 8.7 Duration of ICP fall, minutes: Mannitol:57 ± 31 HTS: 55 ± 32
Carter C. 2017	Case–control study	United States	44 (11 5%NaCl, and 33 23.4%NaCl)	5% NaCl: 55 ± 1623.4% NaCl: 43 ± 17	–	5% NaCl or 23.4% NaCl	–	–	reductions in ICP at 30 min: 5% NaCl: 34 23.4% NaCl: 26 reductions in ICP at 60 min: 5% NaCl: 48 23.4% NaCl: 40 reductions in ICP at and 120 min: 5% NaCl: 46 23.4% NaCl: 30
Schatzmann C. 1998	Clinical trial	Germany	6	–	–	100 ml 10% NaCl	–	–	Relative ICP decrease was 43% [28%-58%] Pressure drop: 18 mm Hg [15–27 mm Hg]

*HTS* Hypertonic saline, *RCT* randomized control trial

### Risk of bias

Table 2 shows the results of the risk of bias assessments using the JBI checklists [18]. Based on our assessments, the most common source of bias in our included RCTs was appropriate reporting blinding methods for both patients and outcome assessors. In one of the cross-sectional studies, dealing with confounding variables was a source of bias. In one quasi-experimental study, there was no control group.

**Table 2:** The results of risk of bias assessments

Study	Q1	Q2	Q3	Q4	Q5	Q6	Q7	Q8	Q9	Q10	Q11	Q12	Q13
Randomized controlled trials													
Francony G. 2008	Yes	Yes	Yes	Yes	No	UC	Yes	Yes	Yes	Yes	Yes	Yes	Yes
Vialet R. 2003	UC	Yes	Yes	UC	No	Yes	Yes	Yes	Yes	Yes	Yes	Yes	Yes
Cottenceau V. 2011	Yes	Yes	Yes	Yes	UC	UC	Yes	Yes	Yes	Yes	Yes	Yes	Yes
Jagannatha AT. 2017	Yes	Yes	Yes	Yes	UC	UC	Yes	Yes	Yes	Yes	Yes	Yes	Yes
Cross-sectional studies													
Ware M. 2005	Yes	Yes	Yes	Yes	No	No	Yes	Yes	–	–	–	–	–
Cheng F. 2018	Yes	Yes	Yes	Yes	Yes	Yes	Yes	Yes	–	–	–	–	–
Case–control study													
Carter C. 2017	Yes	Yes	Yes	Yes	Yes	Yes	Yes	Yes	Yes	–	–	–	–
Quasi-experimental study													
Schatzmann C. 1998	Yes	N/A	N/A	No	UC	N/A	N/A	Yes	UC	–	–	–	–

*UC* unclear, *N/A* not applicable

### Summary of findings

A similar efficacy for mannitol and HTS in patients with sustained ICP was reported in 3 studies [22, 24, 28]. In two studies, the daily ICP burden was significantly lower in the HTS group compared to Mannitol [23, 25]. In Jagannatha et al.’s study, Mannitol and HTS had a similar effect on ICP over 6 days, but an increase in the daily mean ICP was observed after this span which was significant only in the Mannitol group [27]. Regarding the different doses of HTS, Chris Carter et al. in a study published in 2017 reported the same efficacy for 5% and 23.4% NaCl for a sustained ICP > 20 mm Hg [26]. Finally, in Schatzmann et al.’s study infusions of HTS decreased ICP effectively [29].

Regarding mortality, a similar mortality rate between HTS and Mannitol was reported in 3 studies [23, 25, 27]. Also, the duration of ICU or hospital stays was not significantly different between HTS and Mannitol in 2 studies [23, 27]. Finally, the neurologic outcome did not differ significantly between HTS and Mannitol in 3 studies that reported this outcome [25, 27, 28].

### Meta-analysis

**Fig. 2:**
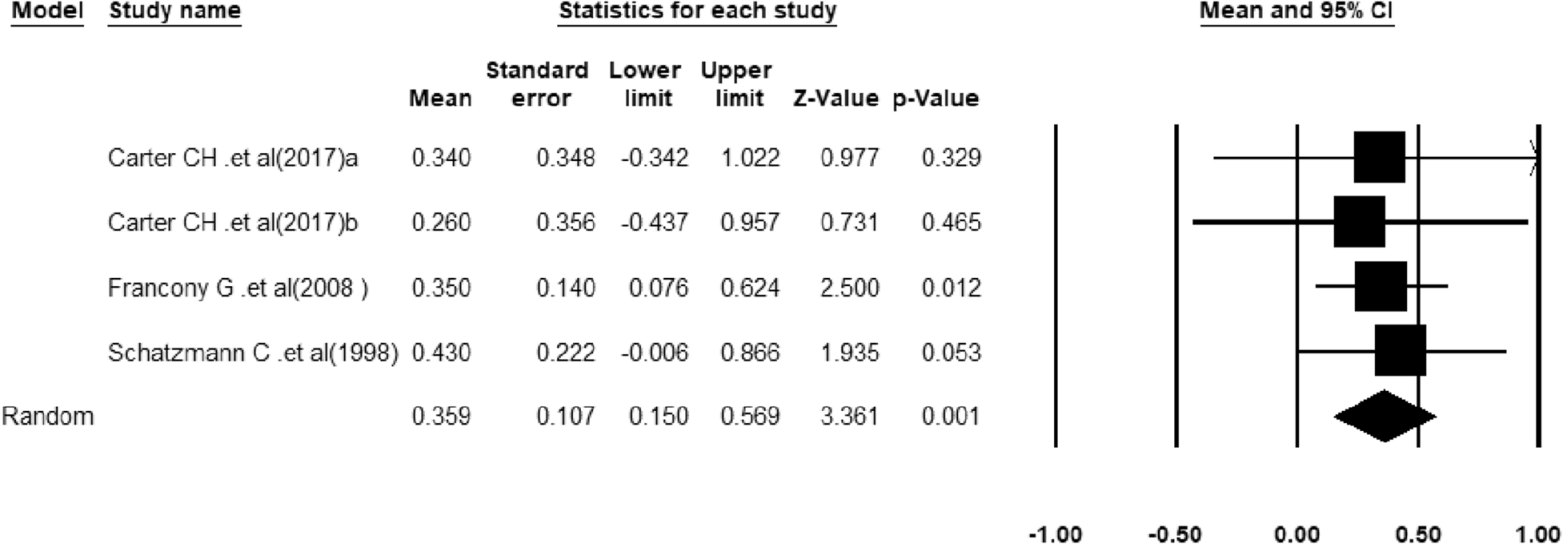
The forest plot for the meta-analysis

**Fig. 3:**
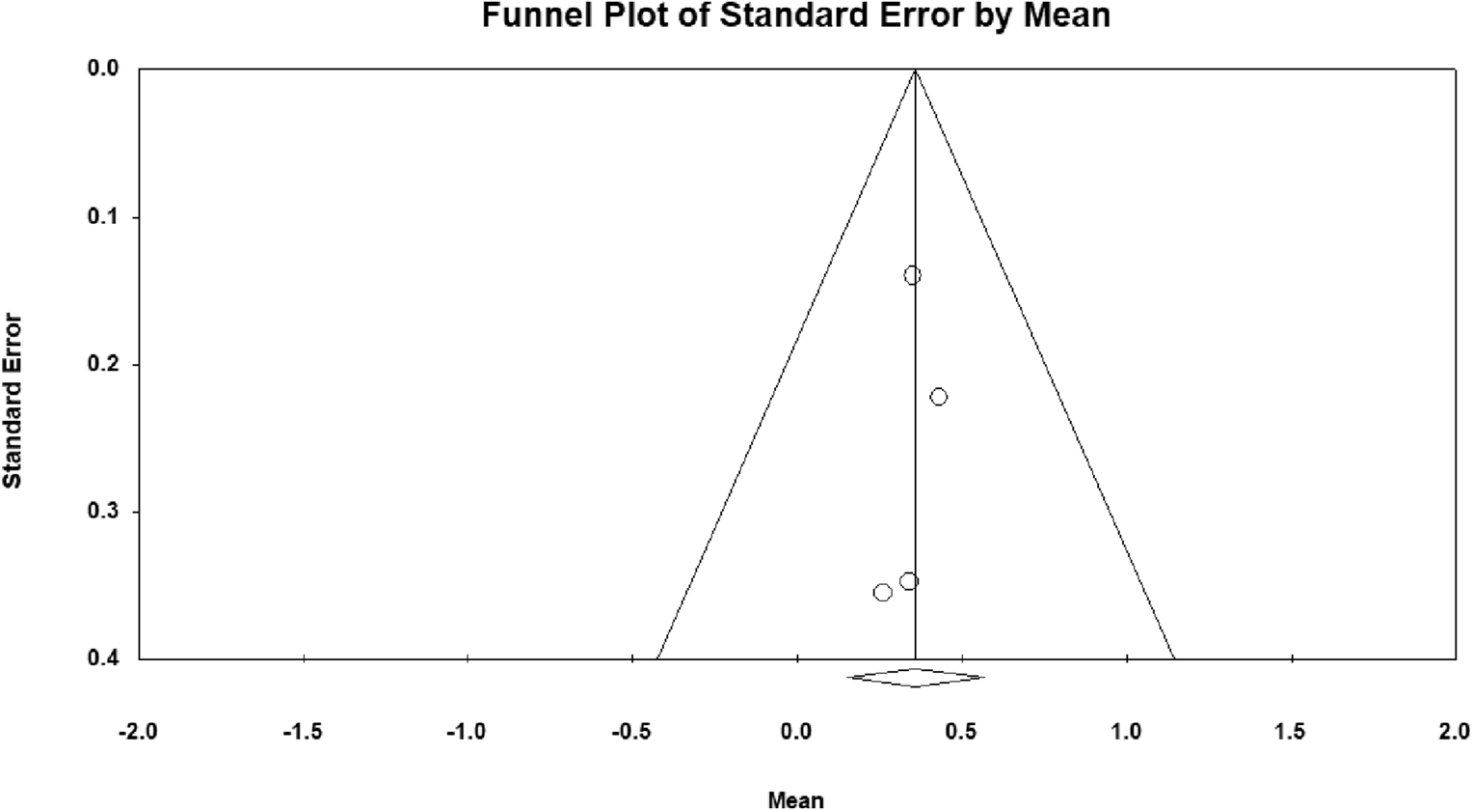
The funnel plot of the studies

A meta-analysis of three studies in which a decrease in ICP was reported in patients receiving HTS was performed. Heterogeneity between studies was not significant (*Q*-value = 0.187, df = 2, *p*-value = 0.98, *I*^2^ = 0.00%). The results of quantitative synthesis reached a 35.9% (95% CI 15.0–56.9) reduction in ICP in TBI patients receiving HTS (Fig. 2). Figure 3 shows the funnel plot to examine the publication bias which was not significant in included studies (*t*-value = 0.38, df = 2, *p*-value = 0.73).

## Discussion

This study considers the effectiveness of HTS in the management of elevated ICP secondary to TBI, lowering mortality rates, and improving neurological outcomes. Based on the available evidence, HTS seems to be efficacious in reducing ICP [22–29]. The results of our meta-analysis reported a 35.9% reduction in ICP in TBI patients with HTS therapy. Also, the neurological consequences [25, 27, 28] and mortality rates [23, 25, 27] do not seem to be significantly different between HTS and Mannitol.

From the mechanism point of view, HTS causes plasma expansion by redistribution of fluid from the extravascular space. Also, the immunomodulatory and anti-inflammatory effects of HTS are reported in previous studies [11, 30–32]. HTS and mannitol share similar mechanisms for lowering elevated ICP by establishing an osmotic gradient across the blood–brain barrier. Increased brain oxygenation is another mechanism suggested by previous studies [33]. Cottenceau et al. reported no effects of mannitol or HTS in boosting the cerebral metabolism, which was assessed by oxygen, glucose, and lactate levels [28]. Also in Jagannatha et al.’s study, sodium level, osmolality, and renal function parameters were comparable between HTS and Mannitol groups [27]. Higher reflection coefficient, effective maintenance of plasma volume, and dehydration of endothelial cells were also suggested as theoretical advantages of HTS [27, 34, 35].

With the implementation of new guidelines since the 1980s, the management of TBI patients appears to be evolving [36]. Despite the absence of Class 1 evidence, ICP monitoring is suggested as a standard clinical observation in TBI patients [37]. A recent scoping review found the evidence regarding the HTS usage in patients with moderate TBI without ICP monitoring inconclusive [38]. We investigated the role of HTS in controlling ICP as the primary outcome and we found that HTS can significantly reduce the ICP in patients with TBI.

Increased ICP is a common life-threatening condition, which is considered the “silent epidemic” [2]. Studies found average ICP in the first two days is an independent predictor of mortality in patients with severe TBI [39]. In addition, the complications of increased ICP include but are not limited to neurological and visual abnormalities, headaches, and nausea [40]. Therefore, this medical and surgical emergency requires prompt recognition and management [41]. Mannitol as a hyperosmolar therapy with wide usage in TBI [42] is considered the gold standard therapy for increased ICP due to TBI, but experts believe it is due to its historical use and not its superiority over HTS [43]. Recent systematic reviews compared mannitol with HS for treating elevated ICP after TBI and found no superiority for none of them [44, 45]. Diuretic properties of mannitol and hypotension reduce the tendency to Mannitol among clinicians. In addition, poor glycemic control with Mannitol also was reported in studies which may affect the overall result of management [27]; therefore, the latest guidelines recommend HTS over Mannitol [46, 47]. The comparison of HTS and Mannitol in this study found HTS as safe and effective as Mannitol; therefore, the choice between them should be based on circumstances, availability, or the clinical situation [48].

Reduction of ICP can be safely achieved with HTS [49]. Francony et al. observed a prolonged duration of ICP reduction in 120 min [22]. Horn et al. also reported that repeated bolus application of HTS could significantly decrease ICP in patients with therapy-resistant elevation of ICP [50]. This finding was in the same line as Munar et al.’s study which found administration of 7.2% HTS effective in reducing ICP [51]. Appropriate reduction in ICP can lead to the prevention of secondary injury and potentially severe complications.

Mannitol is suggested to have a beneficial effect on mortality [52]. The mortality rates were reported in 3 studies. In Cheng et al.’s study and Vialet et al.’s RCT, mortality was not significantly differed between HTS and Mannitol [23, 25]. A comparable difference in 6 months and in-hospital mortality between mannitol and HTS groups was also reported in Jagannatha’s RCT [27]. In this condition and based on the available evidence it seems Mannitol and HTS have no significant superiority over each other in reducing the mortality rate in patients with TBI; however, there is still a need for additional research in this regard [47, 53].

Improvement of neurological outcomes by HTS was reported previously [32]. In our included studies these outcomes were reported in 3 studies. In Vialet et al.’s RCT, neurologic outcomes based on the number of patients with severe Glasgow scale did not differ significantly between Mannitol and HTS [25]. In Cottenceau et al.’s study, the authors did not detect a significant difference in neurological outcome at 6 months, too [28]. Finally, Jagannatha’s RCT also found similar Glasgow scale scores at ICU and hospital discharge, in Mannitol and HTS groups [27], which was similar to the previously mentioned study. The latest Neurocritical Care Society (NCS) guidelines recommended future studies for a comprehensive conclusion in this regard [47].

Studies assessed the efficacy of prehospital HTS resuscitation on neurological outcomes, too. In a RCT conducted by Cooper et al. in 2004, the authors found almost identical neurological function after 6 months, for conventional resuscitation protocols and HTS, which did not support the routine usage of HTS in the prehospital setting [54].

In addition to hyperosmolar therapy, cerebrospinal fluid drainage and barbiturates are also suggested for the management of patients with TBI. Decompressive craniectomy is also suggested as second-line therapy for elevated ICP. A recent Cochrane review assessed the efficacy of this procedure and found it effective in reducing mortality; nevertheless, the authors found the effects on long-term neurological outcomes controversial [7]. Based on the latest guidelines, decompressive craniectomy is only recommended for late refractory ICP elevation [55].

This study was associated with multiple limitations. The limited number of well-designed RCTs, lack of appropriate reports of serum levels of metabolic parameters, such as sodium and glucose, as well as systemic hemodynamics were the main limitations of this study. Also, different reporting methods prevented a comprehensive meta-analysis. We suggest future well-designed prospective multicenter studies with larger sample sizes, appropriate identification and dealing with possible confounding variables, additional cost–benefit analysis, and a longer duration of follow-up—to translate into a long‐term benefit––for reaching more comprehensive and conclusive results on this topic.

## Conclusion

Hyperosmolar therapy with HTS can reduce the ICP significantly, which may lead to the prevention of secondary injury in TBI patients. Based on the available evidence, HTS has relatively similar efficacy to Mannitol, which is considered the gold standard therapy for TBI, in boosting patients’ neurological condition and reducing mortality rates.

## Supplementary Information

**Additional file 1.** Details of search strategies.

## Data Availability

All the supporting data and information are available within the manuscript.
