# Supplementary material for: Hypertonic saline for traumatic brain injury: a systematic review and meta-analysis
Source: Eur J Med Res. 2022 Nov 20;27:254. doi: 10.1186/s40001-022-00897-4 (PMC9677698; doi:10.1186/s40001-022-00897-4)
Supplement: Supplementary file 1 — Additional file 1. Details of search strategies. [file 40001_2022_897_MOESM1_ESM.docx]

## EMBASE

('traumatic brain injury'/de OR 'brain injuries, traumatic':ti,ab OR 'brain lesion, traumatic':ti,ab OR 'brain system trauma':ti,ab OR 'brain trauma':ti,ab OR 'cerebral trauma':ti,ab OR 'cerebrovascular trauma':ti,ab OR 'encephalopathy, traumatic':ti,ab OR 'mild traumatic brain injury':ti,ab OR 'organic cerebral trauma':ti,ab OR 'posttraumatic encephalopathy':ti,ab OR 'traumatic brain injuries':ti,ab OR 'traumatic brain injury':ti,ab OR 'traumatic brain lesion':ti,ab OR 'traumatic cerebral lesion':ti,ab OR 'traumatic encephalopathy':ti,ab) AND ('hypertonic salin normal':ti,ab OR 'sodium chloride'/de OR 'alcathion':ti,ab OR 'bacteriostatic sodium chloride 0.9%':ti,ab OR 'broncho saline':ti,ab OR 'hypertonic lactated saline solution':ti,ab OR 'hypertonic saline':ti,ab OR 'hypertonic saline bath':ti,ab OR 'hypertonic sodium chloride':ti,ab OR 'hypertonic sodium chloride solution':ti,ab OR 'hypotonic sodium chloride':ti,ab OR 'hypotonic sodium chloride solution':ti,ab OR 'natrium chloride':ti,ab OR 'natural saline':ti,ab OR 'normal saline':ti,ab OR 'physiological saline':ti,ab OR 'physiological solution':ti,ab OR 'saline':ti,ab OR 'saline solution':ti,ab OR 'saline solution, hypertonic':ti,ab OR 'salt':ti,ab OR 'sodium chloride':ti,ab OR 'sodium chloride 0.45%':ti,ab OR 'sodium chloride 0.9%':ti,ab OR 'sodium chloride 23.4%':ti,ab OR 'sodium chloride 3%':ti,ab OR 'sodium chloride 5%':ti,ab OR 'sodium chloride solution':ti,ab OR 'sodiumchloride':ti,ab OR 'table salt':ti,ab) AND ('mannitol'/de OR 'acrosmosol':ti,ab OR 'aridol':ti,ab OR 'bronchitol':ti,ab OR 'd mannitol':ti,ab OR 'd-mannitol':ti,ab OR 'dextro mannitol':ti,ab OR 'isotol':ti,ab OR 'manitol':ti,ab OR 'manitol pisa':ti,ab OR 'mannit':ti,ab OR 'mannite':ti,ab OR 'mannitol':ti,ab OR 'mannitol 10%':ti,ab OR 'mannitol 10% with dextrose 5% in distilled water':ti,ab OR 'mannitol 15%':ti,ab OR 'mannitol 15% with dextrose 5% in sodium chloride 0.45%':ti,ab OR 'mannitol 20%':ti,ab OR 'mannitol 25%':ti,ab OR 'mannitol 5%':ti,ab OR 'mannitol 5% with dextrose 5% in sodium chloride 0.12%':ti,ab OR 'mannitol hydrochloride':ti,ab OR 'mannitolum':ti,ab OR 'osmitrol':ti,ab OR 'osmitrol 10% in water':ti,ab OR 'osmitrol 15% in water':ti,ab OR 'osmitrol 20% in water':ti,ab OR 'osmitrol 5% in water':ti,ab OR 'osmofundin':ti,ab OR 'osmohale':ti,ab OR 'osmosal':ti,ab OR 'osmosteril':ti,ab OR 'pearlitol':ti,ab OR 'resectisol':ti,ab OR 'ringer lactate solution'/de OR 'ringer lactate solution':ti,ab OR 'ringer`s lactate':ti,ab OR 'acetated ringer solution':ti,ab OR 'lactate ringer solution':ti,ab OR 'lactated ringer solution':ti,ab OR 'lactated ringer`s':ti,ab OR 'lactated ringers':ti,ab OR 'lactated ringers irrigation':ti,ab OR 'lactated ringer`s solution':ti,ab OR 'lactosol':ti,ab OR 'ringer lactate':ti,ab OR 'ringer lactated solution':ti,ab OR 'ringer saline solution':ti,ab OR 'ringer solution lactated':ti,ab OR 'ringer solution, lactated':ti,ab OR 'glucose'/de OR 'cartose':ti,ab OR 'corn sugar':ti,ab OR 'd glucose':ti,ab OR 'dextro glucose':ti,ab OR 'dextropur':ti,ab OR 'dextrose':ti,ab OR 'dextrose 10%':ti,ab OR 'dextrose 2.5%':ti,ab OR 'dextrose 20%':ti,ab OR 'dextrose 25%':ti,ab OR 'dextrose 30%':ti,ab OR 'dextrose 38.5%':ti,ab OR 'dextrose 40%':ti,ab OR 'dextrose 5%':ti,ab OR 'dextrose 50%':ti,ab OR 'dextrose 60%':ti,ab OR 'dextrose 7.7%':ti,ab OR 'dextrose 70%':ti,ab OR 'dextrosol':ti,ab OR 'glucodin':ti,ab OR 'glucola':ti,ab OR 'glucolin':ti,ab OR 'glucose':ti,ab OR 'glucose hypotonic solution':ti,ab OR 'glucose influx':ti,ab OR 'glucose medium':ti,ab OR 'glucose solution':ti,ab OR 'glucose solution, hypertonic':ti,ab OR 'glutol':ti,ab OR 'glycose':ti,ab OR 'glycovarin':ti,ab OR 'grape sugar':ti,ab OR 'hypertonic dextrose solution':ti,ab OR 'hypertonic glucose solution':ti,ab OR 'hypotonic glucose':ti,ab OR 'hypotonic glucose solution':ti,ab OR 'koladex':ti,ab OR 'saccharum amylaceum':ti,ab OR 'starch sugar':ti,ab OR 'vamin glucose':ti,ab OR 'side effect'/de OR 'side effect':ti,ab OR 'side reaction':ti,ab OR 'effective dose':ti,ab OR 'effective dosage':ti,ab OR 'effective dose (pharmacology)'/exp OR 'ed (drug dose)':ti,ab OR 'ed (effective drug dose)':ti,ab OR 'effective dose (pharmacology)':ti,ab OR 'effective drug dose':ti,ab)

## PubMed

((((((((((((((((((((((((((((((((((((((((((((((((((((((((((((((("mannitol"[Title/Abstract] OR "aridol"[Title/Abstract]) OR "bronchitol"[Title/Abstract]) OR "d mannitol"[Title/Abstract]) OR ("dextro"[All Fields] AND "mannitol"[Title/Abstract])) OR "manitol"[Title/Abstract]) OR ((("mannitol"[MeSH Terms] OR "mannitol"[All Fields]) OR "manitol"[All Fields]) AND "pisa"[Title/Abstract])) OR "mannit"[Title/Abstract]) OR "mannite"[Title/Abstract]) OR ((("mannitol"[MeSH Terms] OR "mannitol"[All Fields]) OR "mannitols"[All Fields]) AND "hydrochloride"[Title/Abstract])) OR "mannitolum"[Title/Abstract]) OR "osmitrol"[Title/Abstract]) OR (((("mannitol"[MeSH Terms] OR "mannitol"[All Fields]) OR "osmitrol"[All Fields]) OR "mannitols"[All Fields]) AND "in water"[Title/Abstract])) OR (((("mannitol"[MeSH Terms] OR "mannitol"[All Fields]) OR "osmitrol"[All Fields]) OR "mannitols"[All Fields]) AND "in water"[Title/Abstract])) OR (((("mannitol"[MeSH Terms] OR "mannitol"[All Fields]) OR "osmitrol"[All Fields]) OR "mannitols"[All Fields]) AND "in water"[Title/Abstract])) OR "osmohale"[Title/Abstract]) OR "osmosteril"[Title/Abstract]) OR "pearlitol"[Title/Abstract]) OR "ringer lactate solution"[Title/Abstract]) OR "Ringer's Lactate"[Title/Abstract]) OR "acetated ringer solution"[Title/Abstract]) OR "lactate ringer solution"[Title/Abstract]) OR "lactated ringer solution"[Title/Abstract]) OR "lactated ringer s"[Title/Abstract]) OR "lactated ringers"[Title/Abstract]) OR ((((("Ringer's Lactate"[MeSH Terms] OR ("ringer`s"[All Fields] AND "lactate"[All Fields])) OR "Ringer's Lactate"[All Fields]) OR ("lactated"[All Fields] AND "ringers"[All Fields])) OR "lactated ringers"[All Fields]) AND "irrigation"[Title/Abstract])) OR "lactated ringer s solution"[Title/Abstract]) OR "lactosol"[Title/Abstract]) OR "ringer lactate"[Title/Abstract]) OR ((("ringer"[All Fields] OR "ringer`s"[All Fields]) OR "ringers"[All Fields]) AND "lactated solution"[Title/Abstract])) OR "Glucose"[Title/Abstract]) OR "corn sugar"[Title/Abstract]) OR "d glucose"[Title/Abstract]) OR ("dextro"[All Fields] AND "Glucose"[Title/Abstract])) OR "dextrose"[Title/Abstract]) OR "dextrose"[Title/Abstract]) OR "dextrosol"[Title/Abstract]) OR "glucola"[Title/Abstract]) OR "Glucose"[Title/Abstract]) OR (((("Glucose"[MeSH Terms] OR "Glucose"[All Fields]) OR "glucoses"[All Fields]) OR "glucose s"[All Fields]) AND "hypotonic solution"[Title/Abstract])) OR "glucose influx"[Title/Abstract]) OR "glucose medium"[Title/Abstract]) OR "glucose solution"[Title/Abstract]) OR "glucose solution hypertonic"[Title/Abstract]) OR "glycose"[Title/Abstract]) OR "grape sugar"[Title/Abstract]) OR "hypertonic dextrose solution"[Title/Abstract]) OR "hypertonic glucose solution"[Title/Abstract]) OR "hypotonic glucose"[Title/Abstract]) OR "hypotonic glucose solution"[Title/Abstract]) OR (("saccharum"[MeSH Terms] OR "saccharum"[All Fields]) AND "amylaceum"[Title/Abstract])) OR "starch sugar"[Title/Abstract]) OR "vamin glucose"[Title/Abstract]) OR "side effect"[Title/Abstract]) OR "side effect"[Title/Abstract]) OR "side reaction"[Title/Abstract]) OR "effective dose"[Title/Abstract]) OR "effective dosage"[Title/Abstract]) OR "effective dose"[Title/Abstract]) OR "Ringer's Lactate"[MeSH Terms]) OR "Glucose"[MeSH Terms]) OR "therapeutic index, drug"[MeSH Terms]) OR "drug-related side effects and adverse reactions"[MeSH Terms]) AND ((((((((((((((((((((((((((((((((((("hypertone"[All Fields] OR "hypertonic"[All Fields]) OR "hypertonics"[All Fields]) OR "muscle hypertonia"[MeSH Terms]) OR ("muscle"[All Fields] AND "hypertonia"[All Fields])) OR "muscle hypertonia"[All Fields]) OR "hypertonicity"[All Fields]) AND "salin"[All Fields]) AND "normal"[Title/Abstract]) OR "Sodium Chloride"[Title/Abstract]) OR "bacteriostatic sodium chloride"[Title/Abstract]) OR ("broncho"[All Fields] AND "saline"[Title/Abstract])) OR "hypertonic lactated saline solution"[Title/Abstract]) OR "hypertonic saline"[Title/Abstract]) OR ((((((("hypertone"[All Fields] OR "hypertonic"[All Fields]) OR "hypertonics"[All Fields]) OR "muscle hypertonia"[MeSH Terms]) OR ("muscle"[All Fields] AND "hypertonia"[All Fields])) OR "muscle hypertonia"[All Fields]) OR "hypertonicity"[All Fields]) AND "saline bath"[Title/Abstract])) OR "hypertonic sodium chloride"[Title/Abstract]) OR "hypertonic sodium chloride solution"[Title/Abstract]) OR "hypotonic sodium chloride"[Title/Abstract]) OR "hypotonic sodium chloride solution"[Title/Abstract]) OR "natrium chloride"[Title/Abstract]) OR "natural saline"[Title/Abstract]) OR "normal saline"[Title/Abstract]) OR "physiological saline"[Title/Abstract]) OR "physiological solution"[Title/Abstract]) OR "saline"[Title/Abstract]) OR "saline solution"[Title/Abstract]) OR "saline solution hypertonic"[Title/Abstract]) OR "salt"[Title/Abstract]) OR "Sodium Chloride"[Title/Abstract]) OR "Sodium Chloride"[Title/Abstract]) OR "Sodium Chloride"[Title/Abstract]) OR "Sodium Chloride"[Title/Abstract]) OR "sodium chloride solution"[Title/Abstract]) OR "sodiumchloride"[Title/Abstract]) OR "table salt"[Title/Abstract]) OR "Sodium Chloride"[MeSH Terms])) AND (((((((((((("traumatic brain injury"[Title/Abstract] OR "brain injuries traumatic"[Title/Abstract]) OR ((((("brain"[MeSH Terms] OR "brain"[All Fields]) OR "brains"[All Fields]) OR "brain s"[All Fields]) AND ((("lesion"[All Fields] OR "lesion s"[All Fields]) OR "lesional"[All Fields]) OR "lesions"[All Fields])) AND "traumatic"[Title/Abstract])) OR (((("brain"[MeSH Terms] OR "brain"[All Fields]) OR "brains"[All Fields]) OR "brain s"[All Fields]) AND "system trauma"[Title/Abstract])) OR "mild traumatic brain injury"[Title/Abstract]) OR ((("organic"[All Fields] OR "organically"[All Fields]) OR "organics"[All Fields]) AND "cerebral trauma"[Title/Abstract])) OR "posttraumatic encephalopathy"[Title/Abstract]) OR "traumatic brain injuries"[Title/Abstract]) OR "traumatic brain injury"[Title/Abstract]) OR "traumatic brain lesion"[Title/Abstract]) OR "traumatic cerebral lesion"[Title/Abstract]) OR "traumatic encephalopathy"[Title/Abstract]) OR "brain injuries, traumatic"[MeSH Terms])
